# Draft Genome Sequences of Two IMP-4-Producing *Escherichia coli* Sequence Type 131 Isolates in Australia

**DOI:** 10.1128/genomeA.00983-15

**Published:** 2015-08-27

**Authors:** Hanna E. Sidjabat, Jennifer Robson, David L. Paterson

**Affiliations:** a University of Queensland, UQ Centre for Clinical Research, Brisbane, Queensland, Australia; b Sullivan Nicolaides Pathology, Brisbane, Queensland, Australia; c Pathology Queensland, Herston, Queensland, Australia

## Abstract

We report the draft genome sequences of two unrelated cases of *Escherichia coli* sequence type 131 (ST131) possessing the carbapenemase gene *bla*_IMP-4_. The *E. coli* ST131 SN5 isolate also possessed *bla*_SHV-12_ and plasmid-mediated quinolone-resistance genes. Wider dissemination of *bla*_IMP-4_ may occur due to the *bla*_IMP-4_-carrying L/M or HI2 plasmids among *E. coli* ST131 isolates.

## GENOME ANNOUNCEMENT

Carbapenem-resistant *Enterobacteriaceae* (CRE) are regarded as an urgent antibiotic resistance threat by the Centers for Disease Control and Prevention (CDC). In the United States, the most common CRE strains produce the KPC beta-lactamase (1). Geographic variations in the type of carbapenemase responsible for CRE exist. The dominance of IMP-4-producing *Enterobacter cloacae* were recently reported in Australia (2). HI2 and L/M plasmids were the two main types of plasmids harboring *bla*_IMP-4_ (2). *Escherichia coli* sequence type 131 (ST131) has been recognized as a highly successful extraintestinal clone of *E. coli* causing urinary tract infections and frequently associated with the production of the extended-spectrum beta-lactamase CTX-M-15 (3).

Two carbapenemase-producing *E. coli* isolates were obtained from patients from two hospitals in Australia. *E. coli* CR48 and *E. coli* SN5 were isolated from urine of an elderly woman and from sputum of an elderly man, respectively. Both *E. coli* isolates were characterized for the genes responsible for the resistance to beta-lactam antibiotics, especially to carbapenems. Initially, both isolates were determined to possess *bla*_IMP-4_ by PCR and classified as *E. coli* ST131 by repetitive sequence-based PCR (repPCR) (DiversiLab, bioMérieux). The two *E. coli* isolates were subjected to whole-genome sequencing to characterize the antibiotic resistance and virulence properties of the isolates.

Whole-genomic DNA of *E. coli* CR48 and SN5 was extracted and prepared using the Nextera XT DNA sample preparation kit and sequenced using the Illumina HiSeq 2000 as previously described (4). *De novo* assembly was performed using CLC Genomics Workbench version 7.5 (CLC Bio, Denmark). The draft genomes of CR48 and SN5 consisted of 5,184,875 and 5,219,367 bp, respectively. Contigs were initially annotated using RAST (http://rast.nmpdr.org). Sequence typing, antibiotic resistance mechanisms, and plasmid Inc types of *E. coli* were determined through http://www.genomicepidemiology.org.

The genome data showed that both *E. coli* isolates were ST131. Both *E. coli* strains also possessed *bla*_TEM-1B_, *sul1*, and *aac(3)-IId*. Additionally, *E. coli* SN5 possessed *bla*_SHV-12_, *bla*_OXA-1_, *qnrA1*, and *qnrB49*. *E. coli* CR48 possessed L/M and FII. Plasmids HI2, HI2A, FII, I1, Col156, and FIB were determined in SN5. The *bla*_IMP-4_ gene was most likely located on the L/M and HI2 plasmids in CR48 and SN5, respectively.

The annotation through RAST identified type 1 fimbriae *fimA-H*, virulence determinants relevant for urinary tract adhesion in both isolates (5). Fimbriae related to the protein secretion system were identified: *htrE* fimbriae cluster, *stf* fimbriae cluster, and colonization factor antigen I fimbriae (CFA/I fimbriae). In addition, fimbriae usher protein StcC was identified. Further, IncF conjugal transfer pili were identified. The cluster responsible for Curli production or type VIII secretion was identified. There were variations of the siderophore enterobactin, siderophore aerobactin, and other hemin transport systems for iron acquisition in the two isolates.

The *bla*_IMP-4_ genes in CR48 and SN5 were both located in the integron class 1. The genetic context of CR48 was *tnpA*|*intI1*|*bla*_IMP-4_|*qacG*|*aacA4*|*catB3*|∆*qacE*|*sul1*|IS*CR1*. Two studies reported the occurrence of IMP-8-producing *E. coli* ST131 in Taiwan and IMP-4-producing *E. coli* ST131 in China (6, 7). These cases of IMP-4-producing *E. coli* ST131 in Australia highlight potential further dissemination of *bla*_IMP-4_ among uropathogenic *E. coli*.

### Nucleotide sequence accession numbers.

This project is registered as BioProject number PRJNA281568 and BioSample numbers SAMN03798494 (*E. coli* ST131 CR48) and SAMN03491847 (*E. coli* ST131 SN5). The draft genomes of IMP-4-producing *E. coli* ST131 CR48 and SN5 have been deposited in GenBank under the accession numbers LFXT00000000 and LFXU00000000, respectively.
